# Population-Based Evaluation of Lumbar Puncture Opening Pressures

**DOI:** 10.3389/fneur.2019.00899

**Published:** 2019-08-16

**Authors:** Feng Wang, Elizabeth R. Lesser, Jeremy K. Cutsforth-Gregory, M. Tariq Bhatti, Khin P. Kilgore, David O. Hodge, Jonathan Graff-Radford, Ronald C. Petersen, David S. Knopman, Michelle M. Mielke, Giuseppe Lanzino, Jaqueline A. Leavitt, John J. Chen

**Affiliations:** ^1^Department of Ophthalmology, Mayo Clinic, Rochester, MN, United States; ^2^Health Sciences Research/Biomedical Statistics and Informatics, Mayo Clinic, Jacksonville, FL, United States; ^3^Department of Neurology, Mayo Clinic, Rochester, MN, United States; ^4^Department of Epidemiology and Department of Neurology, Mayo Clinic, Rochester, MN, United States; ^5^Department of Neurosurgery, Mayo Clinic, Rochester, MN, United States

**Keywords:** lumbar puncture (LP), opening pressure, obesity, idiopathic intracranial hypertension (IIH), mayo clinic study of aging, age, obstructive sleep apnea

## Abstract

**Importance:** Prior studies evaluating opening pressure (OP) have mostly involved lumbar puncture (LP) for diagnosis of neurologic disease or small cohorts of healthy volunteers and therefore the normal OP is not well-defined.

**Objective:** The goal of this study was to establish the normal range of OP in a community-based population using the Mayo Clinic Study of Aging (MCSA) and to evaluate factors that contribute to OP variability.

**Design:** LP OP were obtained from participants aged 32–95 years between 11/1/07 and 10/1/17, as part of routine data collection for the MCSA, a longitudinal, population-based study of residents of Olmsted County, Minnesota.

**Setting:** A longitudinal, population-based study of residents of Olmsted County, Minnesota.

**Participants:** There were 639 participants (56.8% male; 98.5% white) who underwent LP with recorded OP as part of the MCSA.

**Intervention:** LP.

**Main Outcome(s) and Measure(s):** LP OP was recorded along with variables that could possibly influence its variability, including age, body mass index (BMI), and obstructive sleep apnea (OSA).

**Results:** Six hundred thirty-nine participants (56.8% men) underwent LP with recorded OP; average age was 71.0 years (SD 10.9) with a mean BMI of 28.0 (SD 4.6). Mean OP was 155.4 mmH_2_O (SD 41.9) with a 95% reference interval of 82–242 mmH_2_O (range 60–314; Q1, Q3: 124, 182). Increasing age was associated with lower OP (*p* < 0.001), while increasing BMI was associated with higher OP (*p* < 0.001). Twelve (2%) participants had OP ≥ 250 mmH_2_O; they were younger [58.5 (SD 8.2), *p* < 0.001], had higher BMI [33.6 (SD 4.6), *p* < 0.001], and were more likely to have OSA (75%, *p* < 0.001). Among the 79 participants with repeat LPs within 2.5 years, the coefficient of repeatability (CR) was 64.9. Ten (12.7%) had an OP difference ≥50 mmH_2_O between serial LPs.

**Conclusions and Relevance:** This large population-based study showed that LP OP can vary significantly among individuals. Higher OPs were associated with higher BMI and younger age.

## Introduction

Lumbar puncture (LP) opening pressure (OP) is critical to diagnosing conditions of raised intracranial pressure, such as idiopathic intracranial hypertension (IIH) (1), and intracranial hypotension from cerebrospinal fluid (CSF) leak (2). Despite the recognized importance of LP OP, there is significant variability in technique for measuring OP and still some controversy as to the normal range of OP. Many textbooks provide 200 mmH_2_O as the upper limit of normal in adults (3, 4), but more recent studies have suggested that 250 mmH_2_O may be a more appropriate cutoff (3, 5). In addition, multiple studies have shown various factors can influence LP OP, including age, obesity, needle gauge, Valsalva maneuver, and patient positioning (3, 5–9), while other studies have not found these factors to influence OP (3, 10, 11).

Because LP carries some risk of adverse effects, current OP reference ranges are generated from LP performed for diagnosis of neurologic conditions or in small groups of healthy volunteers (3, 12, 13). The Mayo Clinic Study of Aging (MCSA) is a population-based assessment of variables of normal aging in community-situated individuals, a subset of whom volunteer to undergo LP. The goal of this study was to establish the normal range of OP in a community-based population and evaluate factors that contribute to OP variability.

## Methods

### Study Participants and Data Collection

The MCSA is a population-based study in Olmsted County, Minnesota. Details of the MCSA have been published previously (14). In brief, Olmsted County residents 30–95 years of age were enumerated using Rochester Epidemiology Project resources (15). Eligible participants were randomly sampled, in an age-and sex stratified manner, from the population and invited to participate. All participants were invited to undergo a LP with ~17% of those invited agreeing to the LP. LPs were consistently performed in the mid-morning. CSF data were analyzed from the subset of participants who underwent one or more LPs as part of the MCSA from November 1, 2007, to October 1, 2017. Seven patients with a prior history of meningitis were excluded because of concern that this may affect LP OP. No patients in the cohort had a diagnosis of idiopathic intracranial hypertension or a cerebrospinal fluid leak. Factors that might affect OP, including age, sex, body mass index (BMI), and needle gauge were recorded. Age, sex, and BMI were compared between the MCSA participants that opted to have a LP and those that did not. Additionally, anxiety (3, 6), depression, and headache, which anecdotally could increase the risk of Valsalva during LP and falsely increase OP, resulting in increased variability, were also documented. Headache diagnoses were gathered from ICD9, ICD10, and HICDA codes, and anxiety and depression were recorded during clinical visits using self-reported depressive symptoms (Beck Depression Inventory) and anxiety symptoms (Beck Anxiety Inventory). Vascular risk factors, including obstructive sleep apnea (OSA), hypertension (HTN), diabetes mellitus (DM), hyperlipidemia (HLD), and coronary artery disease (CAD), were tallied using ICD9, ICD10, and HICDA codes. A patient was recorded to have OSA, HTN, DM, HLD, or CAD if the initial diagnosis was made during the 10 years before or 1 year after their initial LP. Patients were recorded to have headaches if the initial headache diagnosis was 10 years to 1 day before their initial LP visit in order to avoid capturing post-LP headaches. Some participants received serial LPs over time as part of the MCSA and these participants were used to evaluate repeatability of LP OP.

Study protocols were approved by the Mayo Clinic and Olmsted Medical Center Institutional Review Boards. All participants provided written informed consent to participate in the MCSA and for the LPs.

### Lumbar Puncture

LP was performed by trained teams according to a standardized protocol at Mayo Clinic (Rochester, MN). Participants were placed in the lateral decubitus position with legs in partial extension and a 3.5-inch 20G or 22G spinal needle with a three-way stopcock was inserted into the subarachnoid space. A 550-mm manometer was attached to the stopcock and the column of CSF fluid was allowed to equilibrate for 1–2 min before recording the OP. All procedures were performed in the middle of the morning.

### Statistics

Continuous measures are conveyed as mean (standard deviation [SD]) or median (Q1, Q3), as specified. Categorical measures are summarized as number (percent). Patients' initial LP OP measurements were analyzed to identify an overall 95% reference interval of OPs and 95% reference intervals of OPs per BMI and age category. Ninety five percent reference intervals were determined by the 2.5th percentile as lower limit and the 97.5th percentile as the upper limit. To compare patient characteristics between patients with and without initial OP ≥ 200 or 250 mmH_2_O, Fisher's exact test was used for categorical variables and Wilcoxon rank sum test was used for continuous variables. The Kruskal-Wallis rank sum test was used to compare OP and the absolute differences between OP measurements across consecutive visits per participant amongst categorical variables. Linear regression models were used to evaluate associations of age, BMI, OSA, CAD, and HTN with the natural log of OP (due to the skewed nature of the data) and the absolute differences between OP measurements across consecutive visits per participant. A linear spline model was also fit to the natural log of OP to examine the change in opening pressure between patients <60 years old and ≥60 years old. For models addressing the natural log of OP, estimates of percent change in OP that correspond with the unit change in the independent variables (β) were estimated, using (e^β^-1)^*^100, along with their 95% confidence intervals. Otherwise, linear estimates in the change of absolute differences in OP corresponding to the unit change in independent variables were estimated along with their 95% confidence intervals. Unadjusted and multivariable models adjusted for age and BMI were used to examine relative contributions of OSA, CAD, and HTN to OP variability. Logistic regression models were used to model the relationship between OSA and BMI with the predicted probability that OP would be ≥200 mmH_2_0. Odds ratios [OR] and associated confidence intervals were estimated. A Bland-Altman plot was generated and the coefficient of repeatability (CR) was calculated to visually depict the variability in the differences between OP measurements from consecutive LP visits within 2.5 years of each other. All reported *p*-values are two-sided and evaluated at the 0.05 significance level.

## Results

### Participant Characteristics

**Figure 1:**
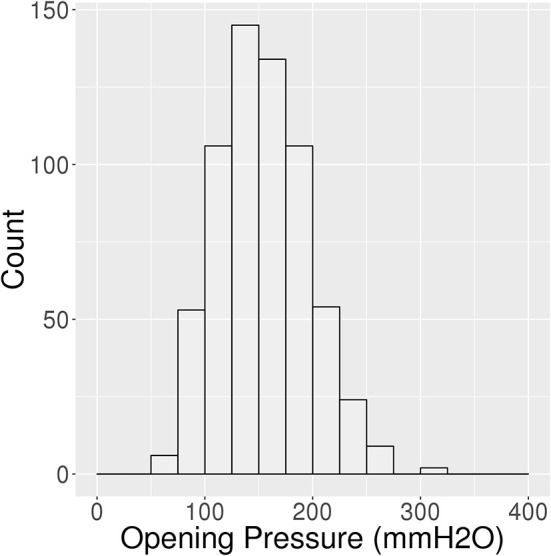
Histogram of opening pressures (*n* = 639).

There were 639 participants (56.8% male; 98.5% white) who underwent LP with recorded OP as part of the MCSA. Average age was 71.0 years (SD: 10.9) with a mean BMI of 28.0 kg/m^2^ (SD: 4.6) (Table 1). Mean OP for all participants was 155.4 mmH_2_O (SD: 41.9; range 60–314; Q1, Q3: 124, 182) with a 95% reference interval of 82–242 mmH_2_O (Figure 1). Only one patient's OP was 60 or less, and she had no clinical or radiographic features of spontaneous intracranial hypotension.

**Table 1:** Demographic and clinical characteristics of all study participants.

**Demographic**	**Statistic**	**(*N* = 639)**
**Age**	*N*	639
	Mean (SD)	71.0 (10.9)
**BMI**	*N*	639
	Mean (SD)	28.0 (4.6)
**Sex**		
Male	*N*%	363 (56.8%)
**Race**	*N*%	
Missing		252
White		383 (99.0%)
Asian		1 (0.3%)
Multiple		3 (0.8%)
**Opening pressure (mmH**_**2**_**O)**	*N*	639
	Mean (SD)	155.4 (41.9)

When comparing the 17% MCSA participants who underwent LP (*n* = 639) with those that did not (*n* = 4,357), there was no significant difference in age (72.8 vs. 73.8, *p* = 0.18) or BMI (27.5 vs. 27.7, *p* = 0.35). Male gender was higher in the patients that elected to undergo a LP (56.8% compared to 49%, *p* < 0.0001).

### Opening Pressure Increases With BMI

**Figure 2:**
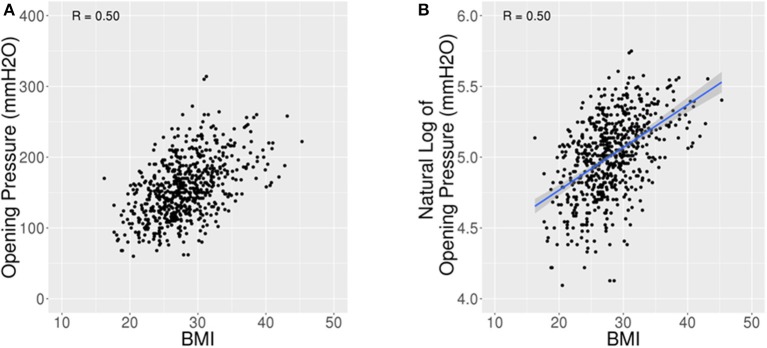
Scatter plots for OP vs. BMI. **(A)** Scatter plot of opening pressure (OP) against body mass index (BMI) showing an increase in OP with higher BMI. **(B)** Scatter plot of natural log of OP against BMI shows a Pearson Correlation of 0.5.

**Figure 3:**
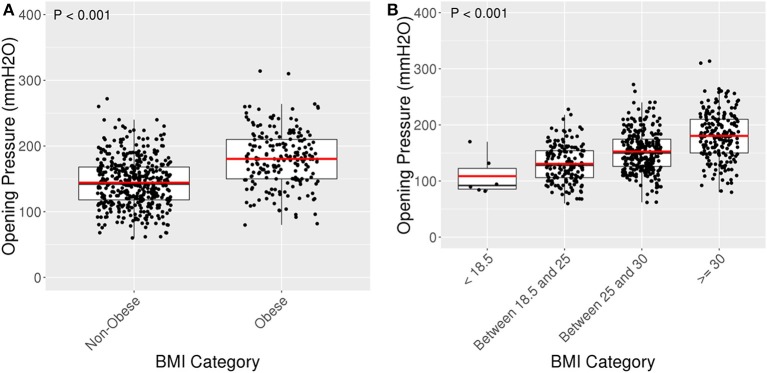
Box plots for OP amongst BMI categories. **(A)** Box plot of OP against non-obese (BMI < 30) and obese (BMI ≥ 30). Obese patients had an average OP of 180.5 mmH_2_O (SD 41.9) compared to an average OP of 144.2 mmH_2_O (SD 36.7) among non-obese patients, *p* < 0.001. **(B)** Jitter plot of opening pressure (OP) compared with BMI categories. Average OP was 108.7 (SD 35.2) for patients with BMI < 18.5, 130.7 (SD 33.5) for BMI 18.5–25, 152.5 (SD 35.9) for BMI 25–30, and 180.5 (SD 41.9) for BMI ≥30, *p* < 0.001.

BMI and OP were positively and moderately correlated with Pearson Correlation (R) = 0.5 (Figure 2). For every five unit increase in BMI, OP is expected to increase by 16% [95% CI: 13.9–18.7%] (*p* < 0.001). The 442 (69.2%) patients with BMI <30 had a lower average OP (Mean: 144.2 mmH_2_O, SD: 36.7) with 95% reference interval of 80.0–221.9 mmH_2_O than the 197 (30.8%) patients with BMI ≥30 (Mean: 180.5 mmH_2_O, SD: 41.9) with 95% reference interval of 101.4–260.0 mmH_2_O (*p* < 0.001) (Figure 3).

Stratifying further, patients with BMI <18.5 had the lowest average OP 108.7 (SD: 35.2), patients with BMI 18.5–25 had the second lowest average OP 130.7 (SD: 33.5), and patients with BMI 25–30 had the second largest average OP 152.5 (SD: 35.9). The largest difference appeared to be between patients with BMI <18.5 and BMI ≥30 (Figure 3).

### Opening Pressure Decreases With Age in Older Patients

**Figure 4:**
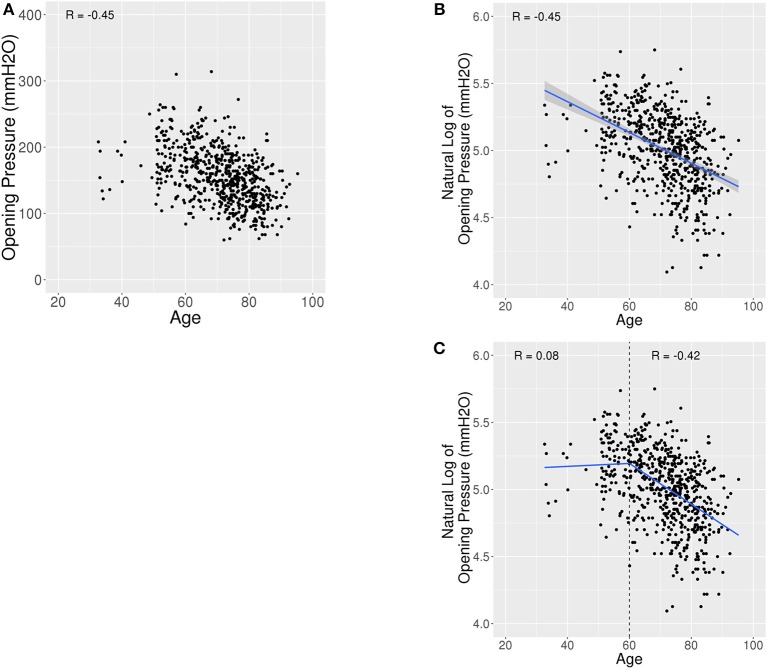
Scatter plots and linear spline model for OP vs. Age. **(A)** Scatter plot of opening pressure (OP) against age showing a lower OP with higher age. **(B)** Scatter plot of natural log of OP against age shows a Pearson Correlation (R) of −0.45. **(C)** Scatter plot of OP against age fit with a linear spline with a knot at age 60. Age was associated with a decrease in OP amongst patients 60 years old or older (*R* = −0.42), but not associated with OP amongst patients younger than 60 years (*R* = 0.08).

**Figure 5:**
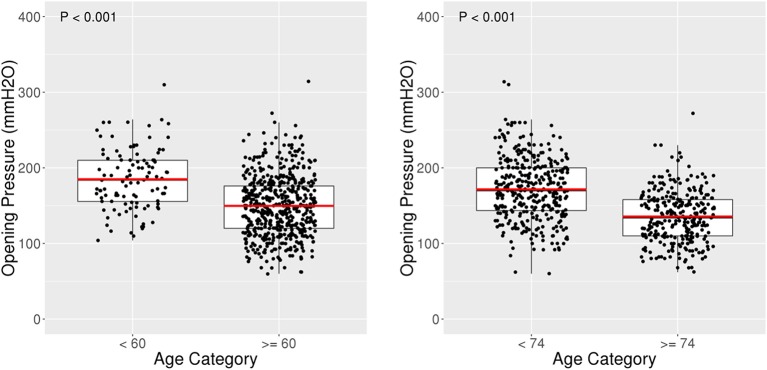
Box plots for OP amongst age categories. Box plot of opening pressure (OP) compared with age categories. Patients <60 years old had an average OP of 184.9 mmH_2_O (SD 40.6) compared to an average OP of 149.7 mmH_2_O (SD 39.7) among patients 60 years or older, *p* < 0.001.

The relationship between opening pressure and age was negative and linear with *R* = −0.45 (Figure 3). The 104 patients <60 years old had an average OP of 184.9 mmH_2_O (SD 40.6) with a 95% reference interval of 115.2–260.0 mmH_2_O. In comparison, 535 patients 60 years or older had a lower average OP of 149.7 mmH_2_O (SD 39.7) with 95% reference interval of 80.7–231.3 mmH_2_O (*p* < 0.001) (Figures 4, 5). Using a spline model, there was a significant negative correlation between opening pressure and age for patients 60 or older (β′ = −13.1% [95% CI: −5.6 to −20.0%], *p* < 0.001) with *R* = −0.42, but there was no significant correlation in patients younger than 60 (β′ = 1.1% [95% CI: −4.7 to 7.2%], *p* = 0.71) with *R* = 0.08 (Figure 4). However, only 16.3% (104/639) of patients were younger than 60 and 2% (13/639) were younger than 50.

### Opening Pressure Associations With Age and BMI

When adjusting OP for age and BMI, both remained significantly associated with OP for the full cohort. For every five unit increase in BMI, OP is expected to increase by 13.6% [95% CI: 11.4–15.8%], and for every 10 year increase in age, OP is expected to decrease by 8.8% [95% CI: 7.3–10.2%] (*p* < 0.001). In this cohort, there was no significant interaction or dependency between age and BMI (β′ = 1.27% [95% CI: −0.42 to 2.98%], *p* = 0.14). Stratifying the sample by age revealed that the association of BMI with OP within patients 60 years and older (β′ = 14.1% [95% CI: 11.6–16.7%], *p* < 0.001) was similar to the effect of BMI in patients 60 years or younger (β′ = 10.2% [95% CI: 6.3–14.2%], *p* < 0.001). Furthermore, BMI remained associated with an increase in OP for both patients younger (*R* = 0.48) and older than 60 (*R* = 0.51). When adjusting for BMI, older age remained associated with a decrease in OP in patients 60 years or older (β′ = −9.99% [95% CI: −12.3 to −7.62%]) and not associated with OP in patients younger than 60 (β′ = 1.61% [95% CI: −4.54 to 8.16%]).

### Opening Pressure Was Not Independently Associated With Other Variables After Adjustment for Age and BMI

Opening pressure was not associated with HLD, DM, headache, depression, anxiety, needle gauge, or sex (Table 2). Patients with OSA (156/639, 24.4%) had higher median OP than patients without OSA (Median: 161 [Q1, Q3: 124, 200] vs. Median: 150 [Q1, Q3: 124, 168], *p* = 0.012). When adjusting for BMI and age, the percent increase of OP in patients with OSA was no longer significant (0.05% [95% CI: −3.98 to 4.24%], *p* = 0.98). Patients with CAD had lower median OP than those without (Median: 148 [Q1, Q3: 120, 170] vs. Median: 157 [Q1, Q3: 127.5, 188], *p* = 0.002), but this was not significant after adjusting for BMI and age (−0.75% [95% CI: −4.62 to 3.29%], *p* = 0.71). Patients with HTN had lower median OP (Median: 150, [Q1, Q3: 124, 180] vs. Median: 160 [Q1, Q3: 130, 186], *p* = 0.029), but this was no longer significant after adjusting for BMI and age (0.86% [95% CI: −2.91%, 4.77%], *p* = 0.66).

**Table 2:** Comparison of variables and their influence on opening pressure.

	***N* (%)**	**Median (Range; Q1, Q3)**	***p*-value**
**Obesity (BMI** **> =** **30)**			<0.001^a^
No	442 (69.2%)	142 (60-272; 118, 168)	
Yes	197 (30.8%)	180 (80-314; 150, 210)	
**Obstructive Sleep Apnea**			0.012^a^
No	483 (75.6%)	150 (60-310; 124, 180)	
Yes	156 (24.4%)	161 (124, 200)	
**Hyperlipidemia**			0.32^a^
No	203 (31.8%)	158 (68-310; 127, 187)	
Yes	436 (68.2%)	150 (60-314; 124, 180)	
**Coronary Artery Disease**			0.002^a^
No	456 (71.4%)	157 (60-310; 127.5, 188)	
Yes	183 (28.6%)	148 (68-314; 120, 170)	
**Diabetes**			0.62^a^
No	346 (54.1%)	151 (68-260; 124, 180)	
Yes	293 (45.9%)	156 (60-314; 126, 182)	
**Hypertension**			0.029^a^
No	266 (41.6%)	160 (60-264; 130, 186)	
Yes	373 (58.4%)	150 (62-314; 124, 180)	
**Headache**			0.070^a^
No	485 (75.9%)	156 (60-314; 124, 186)	
Yes	154 (24.1%)	150 (62-260; 124.5, 170)	
**Depression**			0.15^a^
Missing	44		
No	471 (79.2%)	150 (60-314; 124, 180)	
Yes	124 (20.8%)	158 (62-260; 124, 194.5)	
**Anxiety**			0.99^a^
Missing	44		
No	552 (92.8%)	152 (60-314; 124, 180.5)	
Yes	43 (7.2%)	152 (80-256; 128, 181)	
**Needle Gauge**			0.19^b^
Missing	1		
0	1 (0.2%)	114 (114, 114)	
20	523 (82.0%)	152 (60-310; 124, 182)	
22	114 (17.9%)	160 (68-314; 130.5, 185.5)	
**Sex**			0.99^a^
Male	363 (56.8%)	152 (62-314; 124, 181)	
Female	276 (43.2%)	153 (60-310; 125.5, 182)	
**Race**			N/A
Missing	250		
White	383 (99.0%)	160 (60-314; 129, 188)	
Asian	1 (0.3%)	90 (90, 90)	
Multiple	3 (0.8%)	146 (118-180; 132, 163)	

a Wilcoxon Test;

b Kruskal Wallis Test. Because of the low frequency among racial groups, p-value was not calculated for race.

### Patients With Opening Pressures ≥200 mmH_2_O

There were 101 (15.8%) patients with initial OP ≥200 mmH_2_O. They were younger [Mean: 62.5 (SD: 9.1) vs. Mean: 72.6 (SD: 10.5), *p* < 0.001] and had higher BMI [Mean: 31.8 (SD: 4.40) vs. Mean: 27.3 (SD: 4.26), *p* < 0.001]. More of the higher OP patients had OSA (40.6 vs. 21.4%, *p* < 0.001) and depression (32.6 vs. 18.7%, *p* = 0.003). Fewer had headaches (14.9 vs. 25.9%, *p* = 0.02), CAD (17.8 vs. 30.7%, *p* = 0.01), or HTN (49.5 vs. 60.0%, *p* = 0.049). In logistic regression of OP ≥200 mmH_2_O, both BMI [OR: 3.30, 95% CI: 2.43–4.50] and OSA [OR: 1.70, 95% CI: 1.04–2.80] were significant when included in the same model (*p* < 0.001 and *p* = 0.034, respectively). As such, OSA may contribute to elevated OP independent of BMI at higher OP since the interaction between the two measures was non-significant (*p* = 0.12).

### Patients With Opening Pressures ≥250 mmH_2_O

There were 12 (2.0%) patients with initial OP ≥250 mmH_2_O. They were younger [Mean: 58.5 (SD: 8.2) vs. Mean: 71.3 (SD: 10.8), *p* < 0.001] and had higher BMI [Mean: 33.6 (SD: 4.6) vs. Mean: 27.9 (SD: 4.5), *p* < 0.001]. A majority of them had OSA (75 vs. 23.4%, *p* < 0.001). None of the other variables were statistically significant in this small subset of the cohort. Only 1 (8.3%) had headaches, which was not statistically different from the rest of the cohort (*p* = 0.31). None of the patients with elevated OP were documented to have papilledema on their ophthalmology or optometry examinations, which were done within 5 years of the LP.

### Variability of Serial Lumbar Punctures

**Figure 6:**
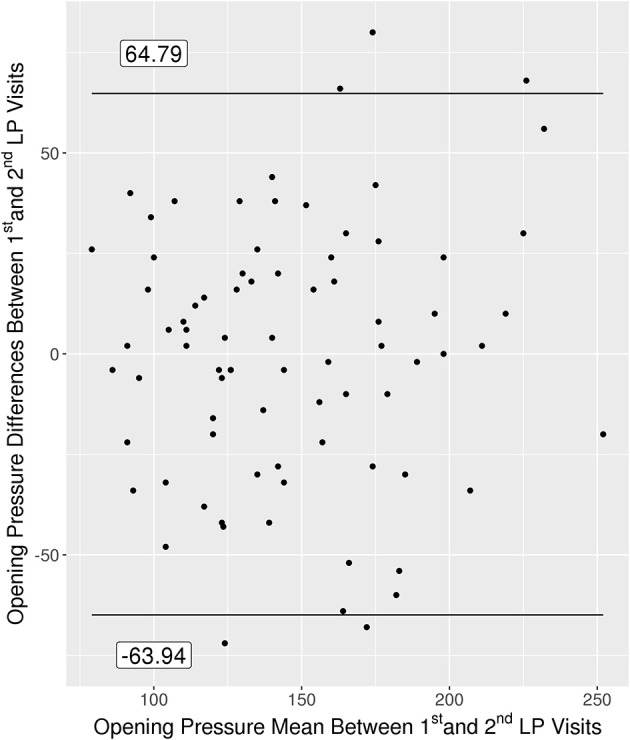
Bland Altman plot for the 79 patients with multiple OPs measured within 2.5 years of each other.

There were 79 patients with two LPs performed within 2.5 years. The differences between the first and second LP OP are shown in the Bland Altman plot (Figure 6). The average difference between the first and second LPs was −0.08 (SD 32.4) mmH_2_O. The coefficient of repeatability (CR) between serial LP OPs was 64.9. Ten of 79 (12.7%) had an absolute OP difference ≥50 mmH_2_O. Two of 79 patients who had an initial OP ≥250 mmH_2_O had subsequent LPs with OPs < 250 mmH_2_O, and one patient of 79 had an initial OP <250 and subsequent LPs with OPs ≥250 mmH_2_O.

Among the patients with repeat LPs, baseline BMI was positively associated with the absolute difference in OPs (*p* = 0.009). For every five unit increase in BMI, the absolute change in OP between the first and second LP visit increased by 7.1 mmH_2_O. None of the other variables examined influenced the variability of serial LPs, including age and OSA.

## Discussion

This is one of the largest studies of consistently-performed LPs in a population-based cohort and therefore provides a reference range for normal OP in adults that is not biased by indication for the procedure. Overall, OP was <200 mmH_2_O in 84.2% of participants and ≥250 mmH_2_O in only 2%. Our data agree with other recent studies suggesting that 250 mmH_2_O is an appropriate cutoff for elevated LP OP (3, 5), but this can be modified slightly based on BMI and age. BMI and age had opposing influences on OP, with obesity associated with higher OP and advanced age associated with lower OP. The upper end of the 95% reference interval for an obese patient is 260 mmH_2_O, while for a non-obese patient it is 222 mmH_2_O. The higher overall reference range for OP in ours and other recent studies compared to some of the prior older studies likely reflects the correlation between BMI and OP and the obesity epidemic in the United States (16).

Recent studies evaluating LPs done predominantly for diagnosis of neurological diseases have also found a correlation between BMI and OP (5, 8, 17). Our population-based study on individuals from the MCSA helps confirm the correlation between BMI and OP. Although there is overlap between OPs in non-obese and obese individuals in our cohort, the average OP was about 25% higher in the obese group. This could influence the interpretation of normal vs. elevated OP. Some studies have not found a correlation between BMI and OP (10, 11, 18); however, these were smaller studies and may not have had the power to detect the correlation. Interestingly, one of the studies showing a lack of association between BMI and OP predominantly included patients with suspected papilledema (11). It is probable that in the pathologic condition of raised intracranial pressure, the disease process trumps the smaller contribution from BMI. Another study showing no correlation with BMI excluded patients with abnormal MR venograms (10). Some patients with higher BMI may have a forme fruste of IIH that accounts for the correlation seen in our population-based study. However, none of the patients in the MCSA with OP >250 mmH_2_O were documented to have papilledema, and the percentage of these patients with headaches was no different than for the rest of the cohort.

It has been speculated that OSA may be the cause of elevated intracranial pressure in some individuals with higher BMI. Our study showed a correlation between OP and OSA, but this correlation was lost when adjusted for BMI in the overall cohort. Other recent studies have not found increased incidence of papilledema in patients with OSA or of OSA in patients with IIH (19–21). Among those with OP >250 mmH_2_O in our study, however, a much larger proportion of them had OSA compared to the rest of the cohort (75 vs. 23.4%), which maintained significance when factoring in BMI. This suggests that OSA may raise OP above the traditional normal range even in the absence of neurologic disease.

In addition to BMI, the other variable that influenced OP was age, which was inversely correlated. Similar to our findings among participants from the MCSA, Fleischman and colleagues found that OP decreased in older age when they retrospectively evaluated more than 12,000 LPs performed primarily for diagnostic purposes (7). They found that the reduction in OP begins at the 6th decade, which parallels our findings from the MCSA where we found a significant correlation between age and OP in patients older than 60 years, but no correlation when evaluated in patients younger than 60 years. The lower OP found in the elderly population may result from decreased cerebrospinal fluid production (22).

Serial LPs were evaluated to determine the repeatability of OP measurements. While the majority of OPs were consistent within individual patients, 12.7% had an absolute OP difference ≥50 mmH_2_O between serial measurements. The overall CR is 64.9 mmH_2_O, which represents where the absolute difference between consecutive OP measurements are expected to lie with a probability of 95%. A difference of 64.9 mmH_2_O could alter the interpretation of normal vs. abnormal OP. Because Valsalva can increase OP by over 2-fold (9), factors associated with a propensity toward Valsalva were examined, including anxiety, depression, and headaches, none of which influenced the variability in the absolute difference of OPs in our cohort. BMI was the only variable associated with an increase in the absolute difference of OPs between consecutive visits. Although speculative, the increased variability seen in obese patients may come from the added difficulty in obtaining a LP in these individuals, which could result in multiple attempts resulting in either inadvertent punctures prior to obtaining the OP or pain that might result in Valsalva. The higher variability in obese patients is important because OPs are often performed in patients suspected of having IIH, a predominantly obese group (23).

Because the MCSA was originally designed to study the elderly population, the majority of patients were older than 60. This is older than the standard demographic for IIH (23), which is the most common cause of raised intracranial pressure, which is a limitation of the study. Among the small number of young patients in the study, a correlation between BMI and OP was maintained while the correlation between age and OP was not. The limited number of younger patients may have contributed to the lack of association between age and OP in the patients younger than 60. However, these findings are supported by other work that included LPs performed primarily for pathologic reasons (7), which suggests this is generalizable to all adults despite the relatively small number of young patients in our study. In addition, if the trend between OP and age documented in patients older than 60 continued at the same rate to all ages, this would lead to a vastly higher reference range for young patients, which has not been noted in clinical practice or in other studies (7). However, a large population-based study of LP OP's of predominantly 20–40 year olds would be ideal to firmly establish the reference range of OP in young adults. Our data do not apply to children, who likely have a slightly higher reference range of OP (24). Another potential limitation is that there were more males in the MCSA that elected to undergo LP, which could possibly introduce bias. However, there was still a large number of women in the study (*n* = 276) and there was no difference in the LP OP between males and females, and therefore this bias unlikely changed the range of OP found in this study. In addition, there was no difference in age or BMI among MCSA participants that elected to undergo LP compared to those that did not. Lastly, the study included predominantly white patients because of the demographics of Olmsted County, Minnesota, and thus may not be generalizable to other races.

This population-based study of LPs provides a 95% reference interval for OP in adults. The study confirms that OP is correlated with BMI and that there is an inverse correlation between OP and age among elderly patients. Although fairly repeatable, some patients can have significant changes in OP on serial LPs, with obesity contributing to the increased variability.

## Data Availability

All datasets generated for this study are included in the manuscript/supplementary files.

## Author Contributions

FW and JC: conception and design of the study, acquisition and analysis of data, and drafting a significant portion of the manuscript or figures. EL, KK, and DH: acquisition and analysis of data, and drafting a significant portion of the manuscript or figures. JC-G, MB, JG-R, RP, DK, MM, GL, and JL: drafting a significant portion of the manuscript or figures.

### Conflict of Interest Statement

The authors declare that the research was conducted in the absence of any commercial or financial relationships that could be construed as a potential conflict of interest.
